# Clinical and Imaging Spectrum of Laser-Induced Maculopathy Caused by Laser Projection Devices

**DOI:** 10.7759/cureus.94678

**Published:** 2025-10-15

**Authors:** Srinivas M Joshi, Siddharth Maanju, Giriraj Vibhute, Guruprasad Ayachit, Apoorva Ayachit

**Affiliations:** 1 Department of Vitreoretina, M. M. Joshi Eye Hospital, Hubli, IND; 2 Department of Ophthalmology, M. M. Joshi Eye Hospital, Hubli, IND

**Keywords:** laser light injuries, laser maculopathy, photic retinopathy, recreational laser injuries, sub-ilm hemorrhage

## Abstract

Purpose: This study highlights the spectrum of fundus and optical coherence tomography (OCT) findings in a cluster of cases of laser-induced maculopathy and their management.

Methods: This single-centre prospective study enrolled 17 eyes from 17 patients diagnosed with laser-induced maculopathy between September 1 and September 30, 2023. Baseline assessments included visual acuity, fundus examination, and OCT features. All patients were followed up at one month and three months to evaluate visual improvement and morphological changes on OCT. The primary outcome measures were final visual acuity and OCT-based morphological changes observed during the follow-up period.

Results: The study included a total of 17 patients, among whom 14 (82.3%) were male and three (17.6%) were female. The mean age was 24.82 ± 4.21 (mean ± SD) years. All patients presented with unilateral ocular involvement. Thirteen eyes (76.4%) had sub-internal limiting membrane hemorrhage (SIH). Of these, eight (47%) underwent surgery and recovered a vision of 20/20. One (5.8%) eye had intraretinal and subretinal hemorrhage and underwent SF6 gas injection. One (5.8%) eye had a macular hole, and two eyes (11.7%) had outer retinal defects on OCT.

Conclusion: This study highlights the clinical and imaging spectrum of lesions in laser-induced maculopathy. There is a lack of awareness about the potentially sight-threatening complications from the use of recreational laser projection devices. Government directives to regulate the use of laser projection devices are needed to prevent loss of vision in children and young adults.

## Introduction

LASER (Light Amplification by Stimulated Emission of Radiation) is a beam of collimated, coherent, unidirectional, and monochromatic light, the energy of which is focused on a small area [1]. Laser use is widespread as a tool for teaching, for decorative light shows, etc. Lasers are used in the form of laser gloves, laser projectors, and hand-held laser pointers. Laser lights are available in different colors, ranging from blue to red. The less expensive lasers, which are also freely available, use a deep-red laser diode of an approximate wavelength of 650 nm. The more expensive ones use a red-orange 635 nm diode, more easily visible because of the greater sensitivity of the human eye at 635 nm. A green laser of 532 nm is a common alternative [2]. The overall incidence of laser-induced injuries to the eyes is on the rise due to the easy availability of laser beam devices. Maculopathy from laser pointers has been described in detail by several studies [3,4]. Herein, we present a series of macular injuries by laser light projectors and decorative lasers during September, a month of festivities in India.

## Materials and methods

This was a single-center study that included patients who presented to the outpatient department (OPD) with unilateral visual symptoms and an antecedent history of laser light exposure to their eyes during September 2023. The study was approved by the Institutional Ethics Committee at M. M. Joshi Eye Hospital, Hubli, Karnataka, India (Approval number: IEC/MMJEI/FEL/2023/FEL1). All patients were informed about the study, and written informed consent was obtained. The study adhered to the tenets of the Declaration of Helsinki. Detailed history about the nature of visual symptoms, i.e., sudden blurring/loss of vision, scotoma, or near vision difficulties, was noted. Patients with systemic illnesses such as diabetes mellitus, hypertension, bleeding disorders, and a history of Valsalva retinopathy were excluded.

A complete ophthalmic examination was done, visual acuity documented in Snellen as well as logarithm of minimum angle of resolution (logMAR) units. A complete anterior and posterior segment clinical examination and ancillary imaging, such as spectral-domain OCT (SD-OCT) (Spectralis HRA2; Heidelberg Engineering GmbH, Heidelberg, Germany), was done in all eyes. Ultrawide field fundus (UWF) photos were taken on Optos Daytona (Optos Inc., Dunfermline, United Kingdom) in all cases. Multicolor imaging (MCI) (Spectralis HRA2; Heidelberg Engineering GmbH) was done in selected cases.

Fundus findings such as intra-retinal hemorrhage (IRH), subretinal hemorrhages (SRH), retinal tears, sub-internal limiting membrane (ILM) hemorrhage, lamellar macular hole, and full-thickness macular hole (FTMH) were noted. OCT features such as outer retinal layer defects, inner and outer lamellar holes, retinal tears, level of retinal hemorrhages (sub-ILM, sub-hyaloid, subretinal, intraretinal), and full-thickness macular holes were noted. Patients having sub-ILM hemorrhage were advised surgery (standard 25-gauge pars plana vitrectomy (PPV)+ ILM peeling) after waiting for a period of two weeks, to allow for spontaneous resolution of haemorrhage. Eyes with subretinal haemorrhage underwent intravitreal sulfur hexafluoride (SF6) gas injection (pneumatic displacement), within three days of laser injury. A patient with a macular hole was planned for surgical management, but the patient refused. Eyes with lamellar holes or outer retinal layer defects were observed.

At the one-month and three-month follow-up, best corrected visual acuity (BCVA) and OCT parameters were assessed. Statistical analysis was done to compare BCVA and OCT findings between baseline and follow-up visits. Quantitative variables were measured in mean ± standard deviation. Qualitative variables were measured in percentages and frequencies.

## Results

The study included a total of 17 patients (17 eyes), of whom 14 (82.3%) were male and three (17.6%) were female. The mean age was 24.82 ± 4.21 (mean ± SD) years. All the patients had unilateral involvement. Mean VA at presentation was 0.81 ± 0.39 log MAR, and the median was 0.77 log MAR. Demographic, clinical, and OCT features and details of intervention are summarized in Table 1. 

**Table 1:** Demographic, clinical and imaging findings of patients at presentation, one month, and three months Normal OCT implies that the foveal contour and retinal layers were normal. ILMP: internal limiting membrane peeling; DSI: days since injury; VA: visual acuity in logMAR units; IRH: intra-retinal haemorrhage; SIH: sub internal limiting membrane haemorrhage; SRH: sub-retinal haemorrhage; SF6: sulfur hexafluoride; RPE: retinal pigment epithelium; EZ: ellipsoid zone; IZ: interdigitation zone; FTMH: full thickness macular hole; OCT: optical coherence tomography; L: left; R: right

Patient number	Age	Eye	First visit			At 1 months		At 3 months	
			Baseline VA in logMAR	OCT features	Intervention	Final VA	OCT features	Final VA	OCT features
Patient 1	23	R	0.6	SIH	ILMP	0	Normal OCT	0	Normal OCT
Patient 2	22	R	0.47	SIH	ILMP	0	Normal OCT	0	Normal OCT
Patient 3	31	R	0.77	SIH	ILMP	0	Normal OCT	0	Normal OCT
Patient 4	32	R	1.07	SIH	ILMP	0	Normal OCT	0	Normal OCT
Patient 5	25	L	0.8	SIH	ILMP	0	Normal OCT	0	Normal OCT
Patient 6	16	R	0.3	SIH	ILMP	0	Normal OCT	0	Normal OCT
Patient 7	24	R	0.6	SIH	ILMP	0	Normal OCT	0	Normal OCT
Patient 8	29	L	1.08	SIH	ILMP	0	Normal OCT	0	Normal OCT
Patient 9	22	R	1.25	SIH+IZ d isruption	Refused surgery	1	SIH+IZ d isruption	0.3	IZ disruption
Patient 10	19	R	1.3	SIH	Refused surgery	0.3	Normal OCT	0.3	Normal OCT
Patient 11	27	R	0.77	SIH	Refused surgery	0.3	Resolving SIH	0.1	Normal OCT
Patient 12	20	R	1.77	SIH	Refused surgery	1.22	Resolving SIH	0.18	Normal OCT
Patient 13	27	L	1	SRH, IRH, EZ+IZ disruption	SF6	0.47	Resolving IRH, EZ+IZ disruption (SRH resolved)	0.3	EZ+IZ disruption
Patient 14	23	L	0.6	FTMH	Refused surgery	0.6	FTMH	0.6	FTMH
Patient 15	25	L	0.48	SIH	Observed	0	Normal OCT	0	Normal OCT
Patient 16	28	L	0.6	Cystic lesion	Observed	0.3	Cystic size reduced to slit	0.18	Normal OCT
Patient 17	29	R	0.3	RPE, EZ, IZ disruption	Observed	0.3	RPE, EZ, IZ disruption	0.3	Normal OCT

**Figure 1:**
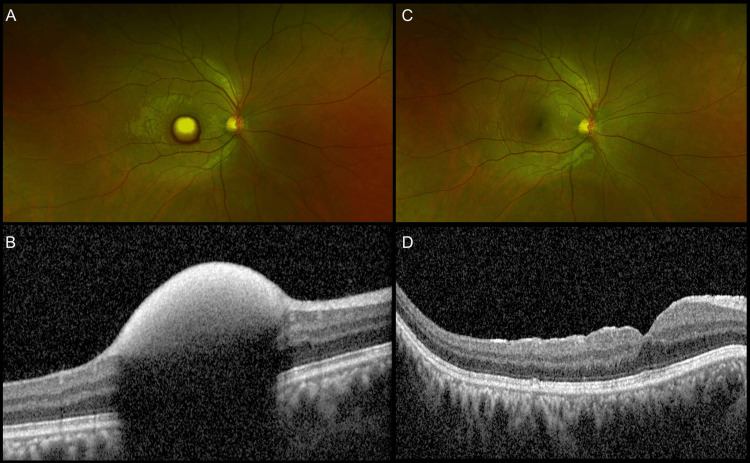
Imaging findings of Patient no. 2 (a) Ultra-wide field (UWF) fundus image of the right eye showing altered sub-ILM hemorrhage; (B) Preoperative OCT image showing the sub-ILM hemorrhage with back shadowing; (C, D) Postoperative UWF image at three months, showing resolution clinically and on OCT. ILM: internal limiting membrane; OCT: optical coherence tomography

Out of the total eyes, 13 (76.4%) eyes had sub-ILM hemorrhage, of which five (29.4%) patients (patient nos. 3, 4, 5, 6, and 7) underwent pars plana vitrectomy (PPV) + ILM peeling + fluid air exchange (FAE), two weeks after presentation. Three (17.6%) patients (patient nos. 1, 2, and 8) had delayed follow-up and were operated on (PPV + ILM peeling + FAE) after one month. Figure 1 shows images of patient no. 2.

**Figure 2:**
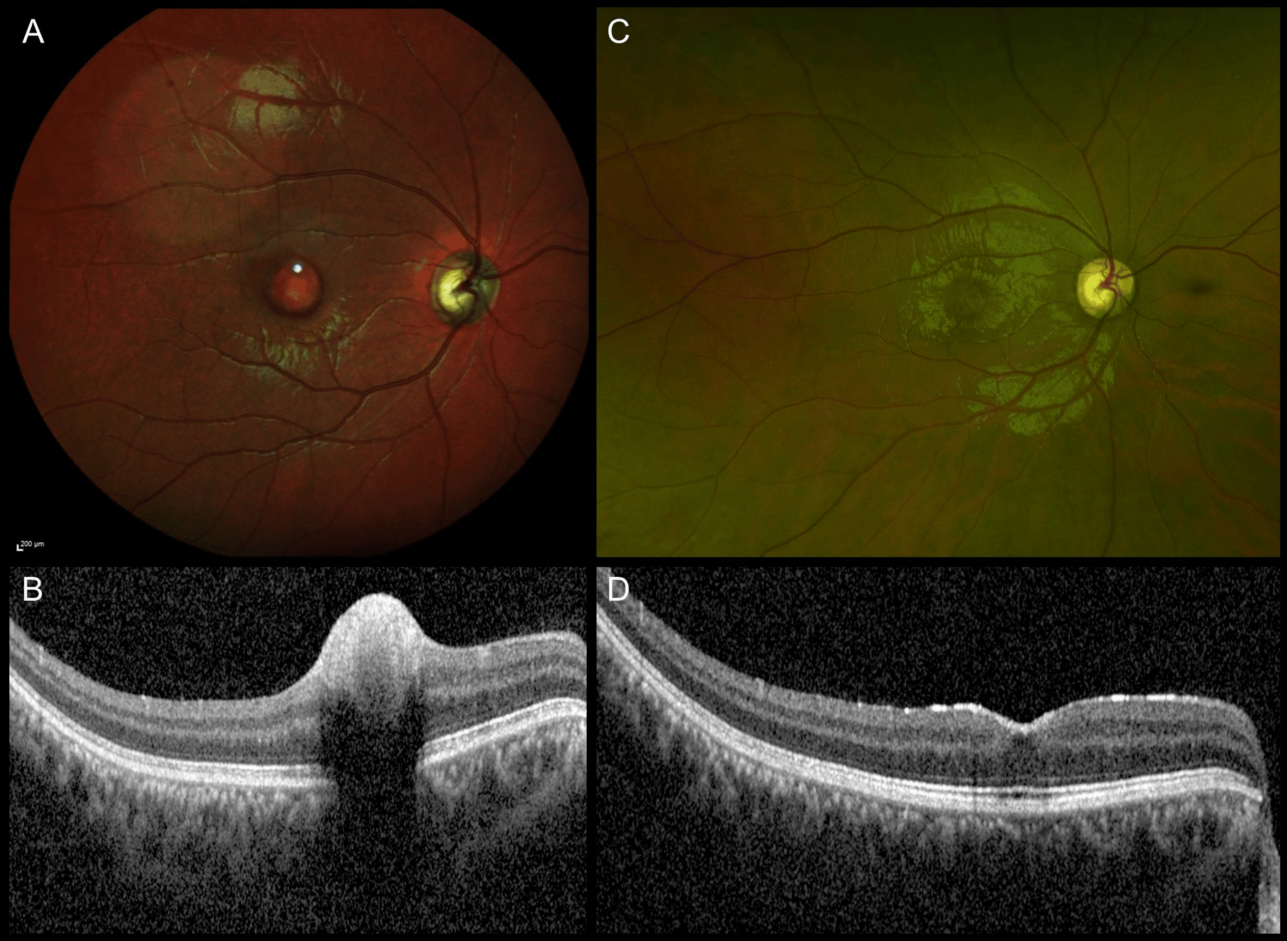
Imaging findings of Patient no. 11 (A) Multicolor fundus image of the right eye at presentation showing sub-ILM hemorrhage; (B) OCT image showing the sub-ILM hemorrhage with back shadowing; (C, D) UWF image at three months showing resolution clinically and on OCT without intervention OCT: optical coherence tomography; ILM: internal limiting membrane; UWF: ultra-wide field

One (5.8%) patient (patient no. 15) was observed, and four (23.5%) patients (patient nos. 9, 10, 11, and 12) refused surgery. All these patients had spontaneous resolution of sub-ILM hemorrhage. Figure 2 shows images of patient no. 11.

**Figure 3:**
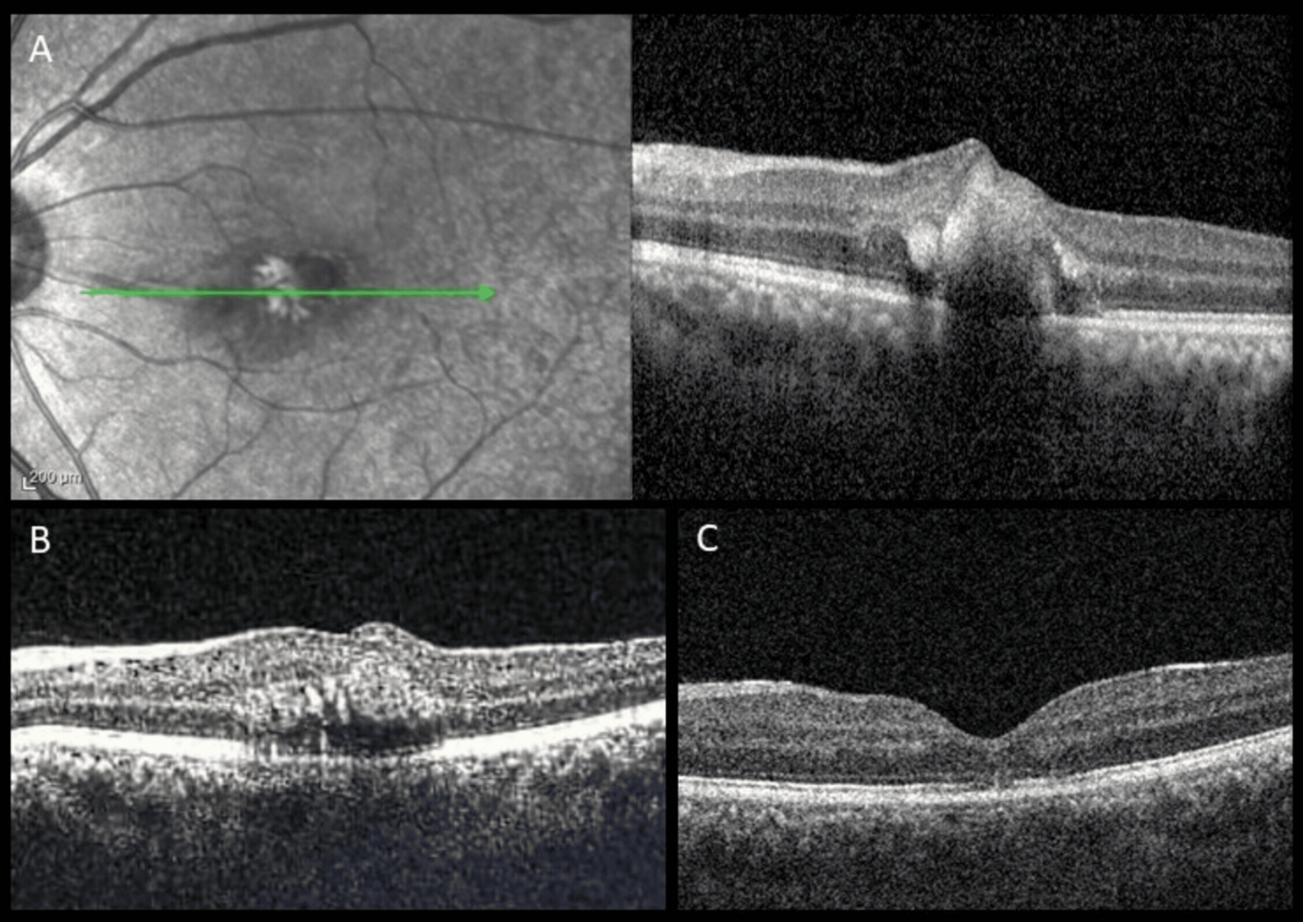
(A) IR image and corresponding OCT line scan through fovea of a patient with subretinal and intra-retinal hemorrhage at presentation; (B) At the one-month follow-up post SF6 gas injection, resolution of subretinal hemorrhage with persistent intraretinal hemorrhage was seen; (C, D) At the three-month follow-up, resolution of intraretinal hemorrhage, with mild thinning of sub foveal ellipsoid zone was seen IR: infrared reflectance; OCT: optical coherence tomography

All eight (47%) patients who underwent surgical management attained visual acuity of 0 logMAR units with complete resolution of hemorrhage. The four (23.5%) patients who refused surgery had visual acuity of 1.14±0.46 logMAR units (mean ± SD) at three months follow-up. Of the 17 eyes, one eye (5.8%) had intraretinal, subretinal hemorrhage and a retinal tear, and was advised to undergo sulfur hexafluoride (SF6) gas injection intravitreally. At one month post-injection, there was complete resolution of the hemorrhage. At three months, OCT showed subfoveal EZ and IZ disruption with a visual acuity of 0.3 logMAR units (Figure 3).

Patient no. 14 presented 20 days after laser light injury with a full-thickness macular hole (basal diameter of 465 µm and minimum linear diameter of 190 µm) and a visual acuity of 0.6 log MAR units. The patient was advised of surgical management for a macular hole, but the patient refused. At three months, OCT showed a flat-open configuration of the macular hole, and the visual acuity remained the same.

**Figure 4:**
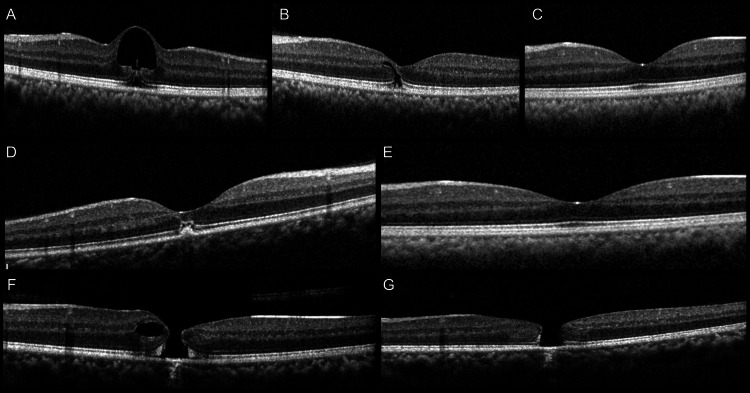
(A) OCT image of a patient with cystic lesion and disruption of outer retinal layers at presentation; (B) At one month, the cystic lesion resolved with a residual hyporeflectivity spanning the middle and outer retinal layers; (C) At three months, there was complete resolution with restoration of foveal contour and outer retinal layers; (D) OCT image of a patient with a spot of sub-RPE hyperreflectivity and disruption of outer retinal layers subfoveally at presentation; (E) Restoration of outer retinal layers at three months; (F) OCT image of a patient with full thickness macular hole and parafoveal cystoid space at presentation; (G) Reduction in cystoid space but persistent full thickness macular hole with flat-open configuration at three months OCT: optical coherence tomography; RPE: retinal pigment epithelium

Patient no. 16 had a cystic lesion with an outer lamellar hole. At the one-month follow-up, the cystic lesion reduced in size to a slit like lesion which involved the inner nuclear, outer plexiform, and outer nuclear layers. Patient no. 17 presented to us with retinal pigment epithelium (RPE), ellipsoid zone (EZ), and interdigitation zone (IZ) disruption. Patient nos. 16 and 17 both had normal OCT at three months with no structural abnormality and had a final visual acuity of 0.18 and 0.3 logMAR units, respectively, at the three-month follow-up (Figure 4).

## Discussion

The month of September in India is a time of religious festivities and is celebrated nationwide with great enthusiasm. It is a time that witnesses elaborate decorations and the extensive use of laser light systems for recreational purposes. With each passing year, the use of these recreational laser projectors has been on the rise [3,4]. These observations led us to compile 17 cases that presented to us in a single month. 

In our study, all injuries were caused by accidental instantaneous exposure to laser lights emitted by laser light projectors at dance parties. This contrasts with literature on laser maculopathy caused by cheap handheld laser pointers [5-7]. Other confounding factors that might cause a similar clinical picture were ruled out, such as valsalva retinopathy, any physical straining or exercise, trauma, and/or systemic illness.

The tissue damage caused by lasers is by either photodisruption (mechanical), photothermal, or photochemical reactions [8]. Photochemical damage is due to photoreceptor protein denaturation, and photodisruption is due to the mechanical impact of the laser energy. Photothermal damage is caused when melanin in the retinal pigment epithelium (RPE) absorbs laser energy, and the temperature in the tissue rises, causing damage [9]. Photoreceptor cells are, in turn, affected because of their proximity to RPE cells [10].

In our study, 14 out of 17 eyes (nearly 82%) had hemorrhage at the macula. This has been postulated due to direct injury to choroidal and retinal blood vessels [11]. This, combined with the phenomenon of instinctive turning of the eye towards the direction of light stimulus when the peripheral retina is stimulated, leads to laser converging on the macula [12]. In our study, all patients with sub-ILM hemorrhage who underwent intervention (surgery or SF6 injection) achieved complete visual recovery to 20/20.

One (5.8%) patient in our study developed a macular hole post laser light injury and was observed, just like in another case report [13]. Another patient who had laser light injury in our study developed a cystic lesion that spontaneously reduced in size, and finally, at the third month, had a normal OCT picture. This pattern of shrinkage of a cystic lesion was also observed in a report that resolved by one year [14]. Patients with structural defects on OCT were followed up without any intervention. At the end of the follow-up, these defects were resolved on their own. Patients who recover to 6/6/N6 generally do not follow up because they would not have further complaints. Hence, we are unable to provide longer follow-up details. We do acknowledge that a longer follow-up is important to look for complications such as epiretinal membrane and macular neovascularization. 

The treatment of patients with steroids in cases of laser light injury remains unproven [15,16]. Hence, in our study, we did not consider the use of steroids and still achieved good visual outcomes.

According to a meta-analysis of patients with laser-induced injury to the eye, 55% of the eyes attained a final vision of 0.96 LogMAR units or better, 36% between 0.176 and 0.698 LogMAR units, and the remaining 9% achieved 1.3 LogMAR units [11]. In our study, 100% had a vision of 0.96 logMAR units or better, 41% between 0.176 and 0.698 logMAR units, and no patient had a vision of 1.3 or higher logMAR units on their last follow-up.

Recreational lasers can have sight-threatening complications. The spectrum of damage by laser injuries includes epiretinal membrane formation, macular cyst, macular hole formation, and even the late sequelae of developing choroidal neovascular membranes years after surgery [17-19].

Due to lack of stringent regulations commercially available high-powered lasers which are incorrectly labelled and have power output far higher than that is indicated on the label are easily accessible [20], and unregulated laser devices where the diffraction filter of the laser devise has been removed to achieve a more focused and flashy beam, thus increasing their damaging potential [4], are a cause of concern. Laser devices with their long range are sometimes intentionally shone at eyes to distract or annoy. These are also directed at aircraft pilots, who may be dazzled or distracted at critical phases of the flight [21]. Apart from this, do-it-yourself videos on YouTube (Alphabet Inc., San Bruno, California, United States) demonstrate the process of making high-powered laser pointers, thus increasing their accessibility.

Shah et al., in early 2024, reported a large series of patients with sub-hyaloid hemorrhage after exposure to high-powered recreational laser projection devices during a festive event [22]. Their series consisted entirely of large sub-hyaloid hemorrhages of a median size of 3 disc diameters (DD). Most of their cases were treated with YAG (yttrium aluminum garnet)-hyaloidotomy. The final follow-up in their series was at one month. There are critical differences between our series and theirs. Our series presents a spectrum of findings such as sub-ILM bleeds (median size of 1 DD), subretinal and intraretinal hemorrhage with retinal tear, full-thickness macular hole, lamellar cystic lesion, and EZ and ELM disruption. Eight (47%) out of 17 eyes underwent PPV+ILM peeling, and one underwent intravitreal injection of SF6 gas. We also present a longer follow-up of three months. Unlike the single-event exposure of all cases in Shah et al.'s report [22], in our study, patients were affected at different time points during the entire month of festivities at different locations, suggesting widespread use of these unregulated devices.

Series such as ours and that by Shah et. al [22] only intensify the magnitude of the problem and show that the occurrences of debilitating laser maculopathy are common, especially in the economically productive demographic. The government needs to have stringent laws for the manufacturing and sale of laser devices. Prohibition of laser devices is needed to reduce the impact of laser-induced injuries, which may otherwise lead to loss of both quality of vision and life in the younger demographic.

Laser light injuries are relatively rare. Our study with 17 consecutive patients highlights how recreational laser projector devices can cause a cluster/epidemic of cases in a single month of festivities. Our series has a relatively large sample size and shows a gamut of functional and morphological damage caused by lasers and further expands on the outcomes of medical and surgical management of these injuries.

## Conclusions

Our case series highlights the growing number of laser light injuries and the severity of the problem they pose. Debilitating laser-induced maculopathy is a common occurrence. The more alarming part of the problem lies in the fact that most victims belong to the economically productive younger demographic, who play a vital role in contributing to society, depriving them of good quality of vision, diminishing their quality of life, and thereby reducing their productivity.

Despite the known harmful effects of laser light exposure, current regulatory measures remain insufficient to curb these injuries. There is an urgent need for stringent laws or even bans on the manufacturing and sale of these devices to prevent their misuse. Without decisive action, these preventable injuries will continue as they have been for many years.
